# Assessment of Patients’ Ability to Review Electronic Health Record Information to Identify Potential Errors: Cross-sectional Web-Based Survey

**DOI:** 10.2196/19074

**Published:** 2021-02-26

**Authors:** Lisa Freise, Ana Luisa Neves, Kelsey Flott, Paul Harrison, John Kelly, Ara Darzi, Erik K Mayer

**Affiliations:** 1 Patient Safety Translational Research Centre Institute of Global Health Innovation Imperial College London London United Kingdom; 2 Center for Health Technology and Services Research / Department of Community Medicine, Health Information and Decision (CINTESIS/MEDCIDS) Faculty of Medicine University of Porto Porto Portugal; 3 Imperial College Healthcare NHS Trust London United Kingdom

**Keywords:** patient portals, electronic health records, patient participation, medical errors, patient safety

## Abstract

**Background:**

Sharing personal health information positively impacts quality of care across several domains, and particularly, safety and patient-centeredness. Patients may identify and flag up inconsistencies in their electronic health records (EHRs), leading to improved information quality and patient safety. However, in order to identify potential errors, patients need to be able to understand the information contained in their EHRs.

**Objective:**

The aim of this study was to assess patients’ perceptions of their ability to understand the information contained in their EHRs and to analyze the main barriers to their understanding. Additionally, the main types of patient-reported errors were characterized.

**Methods:**

A cross-sectional web-based survey was undertaken between March 2017 and September 2017. A total of 682 registered users of the Care Information Exchange, a patient portal, with at least one access during the time of the study were invited to complete the survey containing both structured (multiple choice) and unstructured (free text) questions. The survey contained questions on patients’ perceived ability to understand their EHR information and therefore, to identify errors. Free-text questions allowed respondents to expand on the reasoning for their structured responses and provide more detail about their perceptions of EHRs and identifying errors within them. Qualitative data were systematically reviewed by 2 independent researchers using the framework analysis method in order to identify emerging themes.

**Results:**

A total of 210 responses were obtained. The majority of the responses (123/210, 58.6%) reported understanding of the information. The main barriers identified were information-related (medical terminology and knowledge and interpretation of test results) and technology-related (user-friendliness of the portal, information display). Inconsistencies relating to incomplete and incorrect information were reported in 12.4% (26/210) of the responses.

**Conclusions:**

While the majority of the responses affirmed the understanding of the information contained within the EHRs, both technology and information-based barriers persist. There is a potential to improve the system design to better support opportunities for patients to identify errors. This is with the aim of improving the accuracy, quality, and timeliness of the information held in the EHRs and a mechanism to further engage patients in their health care.

## Introduction

With advancing digitalization and focus on patient centricity, sharing of personal health information with patients has gained increasing importance and prevalence worldwide [1-3]. The UK Department of Health and Social Care [4] has emphasized the need for patients to have access to their records and enhanced control over them. Evidence suggests that sharing personal health information positively impacts quality of care across several domains such as safety and patient-centeredness [5-7].

A potential opportunity to improve patient safety can be realized when patients access and read their health records and identify, and even correct, errors within them [3]. Potential errors include incorrect or missing information concerning administrative details, diagnosis, or treatment, all of which diminish the accuracy, quality, and timeliness of the information held in the medical record, which affects care delivery.

Previous studies have explored the potential of sharing medical records with patients for error correction purposes. Ross and Lin’s [3] review of patient access to medical records indicated that 11 studies reported a facilitation of error correction. However, the studies were based on paper records and the information in 4 studies was anecdotal while that in the other 7 was purely descriptive [3]. Considering electronic health records (EHRs), Bell et al evaluated the impact of an OpenNotes patient reporting tool focused on safety concerns and found that patients and health care partners reported safety concerns in about one-quarter of the reports included in the study, suggesting that a reporting tool could help engage patients as safety partners [8]. However, in order for patients to fully embrace the potential of using EHRs, and particularly to identify and correct potential discrepancies, EHR platforms must support technology acceptance and usability, thereby enhancing a positive user experience and sustained adoption. Previous studies have identified a range of barriers perceived by patients, including attitude and culture challenges [9-11], privacy concerns [12,13], cost concerns [14], poor digital literacy [15], and interface difficulties [11,16]. In a systematic review by Zhao et al (2017), technical or logistical difficulties with the enrollment process (eg, difficult navigation, lack of information technology support) prevented interested patients from completing registration in 15 studies exploring the use of patient portals [15].

Further investigation and understanding of the extent to which patients can identify errors in the EHR will realize current barriers as well as their impact on patients and the safety of their care. The relatively easier access to EHRs compared with paper records might better support patient-initiated correction of errors, although they will likely be presented with a greater granularity of medically related information, which could impact understanding. In addition, patients will need at least some information communication technology skills to navigate an EHR successfully.

Patients’ ability to understand information presented in their EHRs is an essential component for error identification and subsequent correction. If patients cannot interpret information, any proposed advantages of EHR error identification for patient safety will be limited. This pilot study assessed patients’ perceptions of their ability to understand the information contained in their EHRs and the main barriers to their understanding. Additionally, the main types of patient-reported errors were characterized.

## Methods

### Study Design and Setting

A cross-sectional study using electronic survey data collected patients’ use of the Care Information Exchange (CIE). The CIE is a patient-controlled EHR portal in North-West London that allows data sharing between patients and their health care professionals [17]. It was initiated in 2014 at the Imperial College Healthcare National Health Service (NHS) Trust funded by the Imperial College Health Charity [17] and integrates data from sources across health care trusts and care settings. From an organizational perspective, a single patient account is created at the service/department that first registers a patient. However, data from all the departments relevant to each patient’s care are integrated into 1 account. The enrolment of patients in the CIE is optional. Patient information contained in the CIE includes appointment details, test results, care plans, and information on medications. If a patient’s practice has signed up, data such as allergies, medications, and diagnoses will also be visible to them. Clinic letters and discharge summaries can be shared as well. Patients may access their records whenever they wish to review information or when notified about new information such as test results being available. Opportunities and challenges of patient identification of errors within their EHRs were discussed with the National Institute for Health Research (NIHR) Imperial Patient Safety Translational Research Centre’s Research Partners Group, a group of lay partners advising researchers on issues surrounding patient and public involvement and engagement.

### Participants and Sampling

All CIE users accessing their records during the data collection period were invited to participate in this study. Upon logging in, a message signposting the survey was displayed on the CIE webpage to all users during the time of the study (n=682). Patients were asked to follow the link to complete a short survey, which they could complete at a convenient time.

### Data Collection

The survey was open for completion between March 1, 2017 and September 30, 2017. The web-based survey was implemented using Qualtrics (web-based survey software). It contained both structured (multiple choice) and unstructured (free text) questions (Multimedia Appendix 1). Question topics were selected based on information from the CIE standard operating protocol, which was developed from existing evidence about best practice and the necessary characteristics for EHR systems. Responses to the structured questions provided metrics of the patients’ perceived ability to understand their EHR information (ie, “Did you find any information in your record difficult to understand today?”) and identify errors (ie, “When using CIE today, did you notice any errors in your record?”). Responses to unstructured questions supplemented these metrics with qualitative data. Specifically, unstructured questions allowed respondents to expand on the reasoning for their structured responses and provide more detail about their perceptions of EHRs and identifying errors within them. This pilot survey was tested and it underwent the relevant approval process at Imperial College Healthcare NHS Trust (Reference: 675796). There was no direct contact between the survey team and the participants during data collection. Replies to individual questions were not mandated. Patients could complete the survey more than once as it was related to their current visits to the CIE platform.

### Data Analysis

Data were analyzed using Microsoft Excel 2013. Qualitative data were analyzed thematically using the framework analysis method by Ritchie and Spencer [18]. The 6-stage approach was organized as (1) familiarization, (2) identification of a thematic framework, (3) indexing, (4) charting, (5) mapping, and (6) interpretation [18]. A team of 2 researchers (ALN and LF) with previous experience in qualitative research independently and systematically analyzed the data. Subsequently, both discussed their themes together to develop thematic maps for (1) the barriers to understanding EHR information and (2) the kind of errors identified by patients. Themes were supported by quotes from the patients’ free-text survey responses. Data saturation was reached. Results were not returned to participants for comments or feedback. The COREQ (Consolidated Criteria for Reporting Qualitative studies) was used to ensure the study meets the recommended standards of qualitative data reporting [19].

## Results

### Participant Characteristics

The response rate was 23.5% (160/680). A total of 160 patients confirmed that it was their first time responding to the survey, and 210 survey responses were completed. The additional responses may have been made by patients responding more than once as not all respondents stated how many times they had completed the survey. The average time needed to fill in the survey was 6 minutes. The respondents represented a variety of clinical departments, including Oncology, Colposcopy, Early Intervention Services, HIV Service, Interstitial Lung Disease, Neuro-Oncology, and Renal and Rheumatology. Most responses (134/210, 63.8%) were received from services at 1 out of the 3 acute trusts.

### Patients’ Perceptions on Their Ability to Understand the Information in EHRs

Participants were asked whether any information in the record was difficult to understand during their use of the system on the same day. A total of 123 of 210 responses (58.6%) indicated that they did not find it difficult to understand information in the record during their use, while 56 responses (26.7%) indicated at least some difficulties based on the barriers outlined in the section below.

### Barriers to Patients Understanding Their EHR Information

When asked to specify the difficulties they had in understanding information in their EHRs, patients’ replies were classified under 2 broad themes. First, difficulties regarding the information itself and, second, the technology with which the information was relayed to the patients. The presence of difficulties with technology indicates the importance of system usability for its usefulness to patients (Table 1). Concerning the information-related barriers, 2 subthemes of “medical terminology” and “knowledge and interpretation of test results—meaning and significance,” were identified (Table 1). Knowledge of medical terminology was considered a necessary prerequisite to understanding the EHR information and therefore a barrier when lacking (Table 1). Furthermore, the interpretation of EHR information in terms of its meaning and significance could be problematic. This was evidenced by patients’ concerns regarding their inability to understand the meaning of tests, interpret their results, understand their clinical implications, and decide whether any action was required. Patients are unsure about what impact test results might have on their care and what could or should be done as a consequence (Table 1).

The difficulties in understanding information were further perpetuated by technological issues. In terms of the technology-related barriers to understanding EHR information, the EHR portal layout and accessibility was another dominant theme that emerged. Patients reported finding it difficult to find and access information in their EHRs, even when they had previously been notified by the system that their information had been updated. Finally, the presentation of test results (ie, plots instead of raw values) emerged as a theme and another barrier to patients understanding their EHR information, as it impacted what information they were visibly able to extract from the EHR.

**Figure 1:**
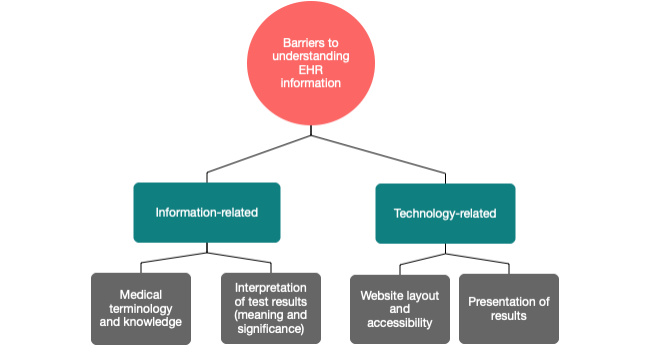
Thematic map of information-related and system-related barriers to patients understanding their records.

In order to provide an overview of both information-related and system-related barriers to patients understanding their records, the themes derived from the survey (as well as the interactions between them) were used to build a thematic map (Figure 1).

**Table 1:** Barriers to patients understanding their electronic health record information.

Barriers, themes		Illustrative quote
**Information-related barriers**		
	Medical terminology	…*Plain English needed against various medical terms/acronyms used.* [Patient ID 1]
	Knowledge and interpretation of test results	…*I do not understand […] how worried I need to be when test results are out of range.* [Patient ID 204] *…You obviously need reasonable medical knowledge to understand results.* [Patient ID 89] *…All test results should have an information sheet attached explaining in layman's terms what it means and if any action is required.* [Patient ID 94]
**Technology-related barriers**		
	Portal layout and accessibility	…*Having received an email stating that my records have been updated, I cannot find the stated change once logged in.* [Patient ID 136] *…I do not know how to access information correctly.* [Patient ID 81] *…I received an email telling me that radiology data had been uploaded to my account, but I was not able to find it. It would have been easier if there had been a path finding link.* [Patient ID 146]
	Presentation of results	…*I would prefer to see [actual] numbers not […] graphs for blood tests.* [Patient ID 100]

### Types of Errors Identified by Patients Within Their EHRs

**Figure 2:**
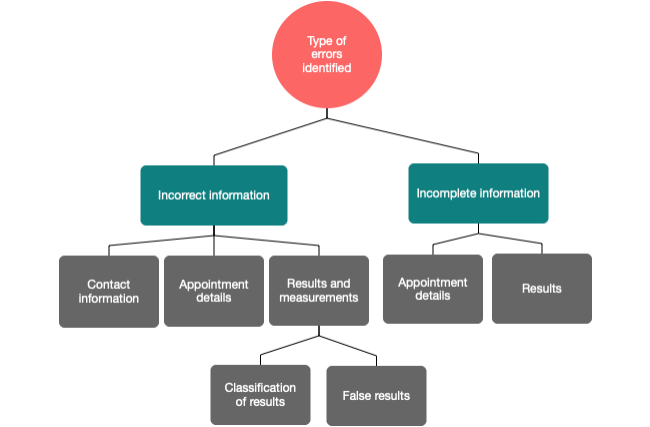
Types of errors identified by patients within their electronic health records.

While the majority of the responses did not identify any error in the EHRs (160/210, 76.2%), 26 responses (12.4%) did indicate noticing some form of error in their records, while 24 responses showed no replies to that question. From the free-text responses, 2 main information-based themes were identified as accounting for the errors patients identified within their EHRs: (1) incorrect and (2) incomplete information (Figure 2). Incorrect information included the subthemes of contact information, appointment details, and results and measurements. Moreover, errors in results and measurements were further divided into the classification of results and measurements and entirely false results and measurements. A detailed description of the errors identified, evidenced by participants’ quotes, is provided in Table 2. The subthemes of incomplete information included information on appointment details and results. Patients commented that not all their appointments or results were visible on the system. While most patients appeared to have at least some information with respect to their appointments on CIE, for some, no information at all regarding their results were accessible, thereby limiting the CIE usefulness for the patient.

**Table 2:** Types of errors identified by patients within their electronic health records.

Themes	Illustrative quotes
Contact information (incorrect information)	…*Incorrect address*. [Patient ID 147 and Patient ID 163]
Appointment details (incorrect/incomplete information)	…*Not all my appointments are accurate.* [Patient ID 180] *…My appointment admission and discharge times are not accurate* [Patient ID 140] *…Not all my appointments are recorded so I can't rely on this so I have to keep a separate record myself.* [Patient ID 180]
Results and measurements (incorrect/incomplete information)	…*An x-ray scan […] was recorded as a CT scan.* [Patient ID 186] *…I had an HRCT scan which had been recorded as an X-ray.* [Patient ID 3] *…No information shown on blood test results or anything other than hospital appointments.* [Patient ID 144] *…Blood glucose of another patient maybe with the same name seems to be there.* [Patient ID 199]

## Discussion

### Summary of the Key Findings

The majority of the survey responses (123/210, 58.6%) reported no problems in understanding EHR information. However, for those reports that did, several difficulties seemed to exist that created barriers to the understanding. These were identified as relating to the information presented (terminology and significance) or to technology-based or system-based issues (layout and result presentation). Furthermore, patients recognized issues of incorrect or incomplete information in a range of aspects, including contact information, appointment details, and results and measurements.

### Findings as Compared With Previous Studies

In a high percentage of responses, patients were able to understand their EHR information. These results are consistent with the findings of a previous observational study assessing patients’ preferences and perspectives in accessing their records, in which 70.8% of the participants found their records easy to understand [20]. In previous studies, patients reported that they understand notes and that reading notes helps them remember next action points such as tests and referrals [8,21], enables timely follow-up of results, supports family or friend care partners with information [8,21-23], and creates a new mechanism for patients to identify documentation errors [24,25].

The likelihood of patients using EHRs and understanding the information is linked to their digital health literacy, defined as “the ability to search, locate, understand, and use health information through electronic resources and use this knowledge to resolve health-related problems” [26]. Digital health literacy can contribute to more informed decision making and potentially improve health outcomes [27]. Previous studies found that patients with higher digital health literacy levels have a higher likelihood of being a portal user [28-31]. Holt et al (2019) actually suggest that information about patients’ health literacy may provide a better understanding of patients’ reasons for not using digital health services, rather than the sociodemographic data [31]. However, similar to other digital health technologies, providing patients with access to correct errors can also increase health inequities, that is, widen the “digital divide” [32]. While patients with higher level of education and better health literacy may want to get more involved in their health care decision making, patients who are less educated may feel that they will not understand the information or may also feel that their doctors know what is best and be less inclined to get involved [33]. Therefore, realizing the potential of patients correcting errors in their EHRs will require broad outreach, engagement, and training for a diverse group of patients of various ages, races/ethnicities, and educational and health literacy levels [34].

As previously found in other studies [35-38], one of the main barriers to understanding medical information was medical terminology and jargon. Acronyms can vary greatly between individuals and specialties, and their understanding can be confusing for clinicians and even more for patients [20]. Strategies to improve readability include efforts to keep usage of terminology consistent [20], minimization of the use of jargon in clinical notes, as well as use of dictionaries as supporting documents in medical records. Other strategies include providing adequate support and training for patients and activities aiming to improve health literacy, that is, patients’ knowledge and ability necessary to understand and act upon health information [39].

As in this study, technology-related issues have previously been identified as inhibiting patients’ use of health records [40]. The navigation of the system as well as the ease of use have been previously criticized by patients in regard to their EHRs [40]. These findings are consistent with the results of this study showing that patients’ ability to use their EHRs effectively is related to the subthemes “user-friendliness” and “website layout.” The technology-related barriers are likely to further perpetuate the identified information-related barriers as the way medical information is presented is known to influence patients’ ability to interpret its significance and the actions needed as a result [40,41]. There is an opportunity for EHRs to support understanding of health information through user-centered designs in their presentations and provision of linked and easily accessible additional information, to support interpretation of the information shared and to provide guidance for patients on how to use this information for their self-care.

Patients and families have unique knowledge about themselves and their own health care, and their reports have potential for improving both individual and organizational safety [34]. The types of errors identified in our study and in those by Mossaed et al in 2015 and Bell et al in 2020 were similar, including missing test results, medications, and wrong date of birth [20,34]. In our study, the potential severity and impact of the different error types identified varied. While a misclassification of a result (eg, “An x-ray scan […] was recorded as a CT scan”) may be inconvenient for finding it on the CIE, this does not necessarily directly pose a threat to patient safety. However, test results from another patient may directly affect future care provision by providing false data to health care professionals and posing safety risks. Hence, as suggested by Bell et al, patients engaging with their EHRs to monitor its contents and flag up potential inconsistencies could enhance quality of care further down the line and offer an opportunity for patients to engage with their health care [7].

In our study, 12.4% (26/210) of the patients identified errors in their medical records. This value is slightly higher than that observed in similar studies published previously, where 7.7% of the patients reported finding errors in their records [20]. In a recent study by Bell et al in 2020, more than 1 in 5 patients perceived mistakes in their notes, with older patients and those with poorer health twice as likely to identify serious errors, suggesting that note sharing may have particularly important safety implications for those groups [34]. As no information on the actual error rate in the patient-controlled EHR was accessible because of patient privacy reasons, the interpretation of the patient-reported error detection percentage could not be explored further.

It is important to note that some patient-reported errors may refer to disagreements between providers and patients and may not necessarily be errors [34]. However, previous evidence suggests that the errors described by patients as very serious usually appeared to have relevant clinical implications [8,34,42], and therefore, patients perceived and reported that error rates remain central to partnering with patients toward the successful and sustainable patient engagement with their EHRs. Future investigations of errors or inconsistencies as identified by patients in contrast to health care professionals may allow for further estimation of the impact of patient EHR review on safety and quality of care.

Consistent with UK policy guidance emphasizing the need to share health information with patients [4,43], a recent initiative has focused on outpatient clinic letters and the style and language used within them to be better “directed” at patients and thereby written in appropriate language [44]. This initiative highlights the political drive to overcome some of the information-related and knowledge-related barriers to understanding information. These approaches may also help mitigate some barriers inhibiting error identification by patients, thus enhancing the effectiveness of future patient-initiated error identification. In addition, receiving clinic letters almost immediately gives patients the means to keep a convenient record of the letters and to ensure that actions advised in these letters are followed through.

### Strengths, Limitations, and Future Work

There are several strengths in this study. The triangulation of interpretations among researchers with expertise in qualitative research, clinical research, and cognitive science resulted in a depth of knowledge and inclusion of different perspectives in this study. Another strength of this study was the diversity in the participant sample, including participants from a range of departments. To ensure the quality of this work, qualitative data were handled with reference to the COREQ checklist, according to best practice recommendations [19]. In order to keep the length of the survey short, encourage responses, and minimize the risk of patient reidentification, the information collected on participant demographics, characteristics, and context of their CIE access was limited. A few demographic factors have been previously identified as playing a role in a patient’s ability to understand the information contained in their EHRs, including female gender, younger age (<60 years), and having a higher level of education [20]. Future work will need to investigate how a wider range of demographic, contextual, and social factors, as well as patient activation and health literacy influence patients’ perspectives and abilities to identify errors in their EHRs, thereby allowing planning and implementation of quality improvement work to support its posited benefits without causing any disadvantage to any patient group.

Respondents could complete the survey more than once, thereby limiting the aspects of the data interpretation. However, exclusion of responses would have significantly reduced the scope of the data, and we wanted to be sure to capture and use all respondents’ feedback, especially when they took the time to provide feedback multiple times. Equally, over time, an individual's feedback could be perceived to become more insightful as their experience with using the patient portal increased. As no information on the scope of errors existing in the questioned patients’ records was available, interpretation of the error detection percentage is limited. Future investigation of errors or inconsistencies as identified by patients in contrast to health care professionals may allow for further estimation of the impact of patient EHR review on safety and quality of care. Additionally, it would be important for future research to examine associations between patient-reported errors and safety outcomes [34]. Finally, future work should include methodologically robust quantitative studies focusing on quantifying how different factors influence both the public willingness and ability to correct errors in their EHRs.

### Conclusions

EHR systems have the potential to support patient engagement in health care by providing personal health information to patients. While the majority of the patients reportedly understand the information contained within their EHRs, technology and information-based barriers persist. Future implementation of such systems must consider supporting patients in the interpretation of the information presented, in what concerns both terminology and significance, and partner with a diverse group of patients to co-design solutions with appropriate usability. Additionally, organizations will need to develop systematic mechanisms for triaging and responding to patient-reported errors, particularly as EHR transparency increases. At a moment when public demand for data is growing, along with a greater awareness of health care data ownership, these barriers must be addressed and their solutions incorporated in health information systems that support patients and their care providers to together improve patient safety.

## Appendix

Multimedia Appendix 1 Patient survey.

## Abbreviations

CIE: Care Information Exchange
COREQ: Consolidated Criteria For Reporting Qualitative Studies
EHR: electronic health record
NHS: National Health Service
NIHR: National Institute for Health Research
