# Supplementary material for: Assessment of Patients’ Ability to Review Electronic Health Record Information to Identify Potential Errors: Cross-sectional Web-Based Survey
Source: JMIR Form Res. 2021 Feb 26;5(2):e19074. doi: 10.2196/19074 (PMC7954650; doi:10.2196/19074)
Supplement: Multimedia Appendix 1 [file formative_v5i2e19074_app1.pdf]

Supplementary file 1 – CIE Survey

Question 1: How many times have you completed this survey in the past?
- I have never completed this survey
- 1
- 2
- 3
- 4
- More than 4 times

Question 2: When using the CIE today, did you notice any errors in your record?
- No, I did not notice any errors
- Yes, I did notice major errors - please specify *(free text response)*
- Yes, I did notice minor errors - please specify *(free text response)*

Skip To: Question 3 If When using the CIE today, did you notice any errors in your record? = No, I did not notice any errors

If you have noticed errors in your record and have not already reported them, then we urge you to contact your GP practice.

Question 3: Did you find any information in your record difficult to understand today?
- No
- Yes - please specify *(free text response)*

Question 4: Did you have any queries after viewing your record today, and did you contact anyone to discuss?
- No, I had no queries
- Yes, I had a query and I could not find anyone to speak to about it - please give details of your query *(free text response)*
- Yes, I had a query and I asked my doctor about it - please give details of your query *(free text response)*
- Yes, I had a query and I spoke to someone other than my doctor - please give details of your query *(free text response)*

Question 5: Please tell us where you were signed up for a CIE account?
- Early Intervention Service A
- Early Intervention Service B
- HIV
- Interstitial Lung Disease
- Neuro-oncology
- Colposcopy
- Oncology
- Rheumatology
- Renal
